# Development and content validity of a patient reported outcomes measure to assess symptoms of major depressive disorder

**DOI:** 10.1186/1471-244X-12-34

**Published:** 2012-04-25

**Authors:** Kathryn Eilene Lasch, Mariam Hassan, Jean Endicott, Elisabeth Carine Piault-Luis, Julie Locklear, Marcy Fitz-Randolph, Sanjeev Pathak, Steve Hwang, Kasey Jernigan

**Affiliations:** 1 Adelphi Values, Boston, MA, USA; 2 AstraZeneca Pharmaceuticals LP, Wilmington, DE, USA; 3 Columbia University, New York, NY, USA

**Keywords:** Major Depressive Disorder, Patient-Reported Outcomes, Quality of life, Questionnaire development

## Abstract

**Background:**

Although many symptoms of Major Depressive Disorder (MDD) are assessed through patient-report, there are currently no patient-reported outcome (PRO) instruments that incorporate documented evidence of patient input in PRO instrument development. A review of existing PROs used in MDD suggested the need to conduct qualitative research with patients with MDD to better understand their experience of MDD and develop an evaluative instrument with content validity. The aim of this study was to develop a disease-specific questionnaire to assess symptoms important and relevant to adult MDD patients.

**Methods:**

The questionnaire development involved qualitative interviews for concept elicitation, instrument development, and cognitive interviews to support content validity. For concept elicitation, ten MDD severity-specific focus group interviews with thirty-eight patients having clinician-confirmed diagnoses of MDD were conducted in January 2009. A semi-structured discussion guide was used to elicit patients' spontaneous descriptions of MDD symptoms. Verbatim transcripts of focus groups were coded and analyzed to develop a conceptual framework to describe MDD. A PRO instrument was developed by operationalizing concepts elicited in the conceptual framework. Cognitive interviews were carried out in patients (n = 20) to refine and test the content validity of the instrument in terms of item relevance and comprehension, instructions, recall period, and response categories.

**Results:**

Concept elicitation focus groups identified thirty-five unique concepts falling into several domains: i) emotional, ii) cognitive, iii) motivation, iv) work, v) sleep, vi) appetite, vii) social, viii) activities of daily living, ix) tired/fatigue, x) body pain, and xi) suicidality. Concept saturation, the point at which no new relevant information emerges in later interviews, was achieved for each of the concepts. Based on the qualitative findings, the PRO instrument developed had 15 daily and 20 weekly items. The cognitive interviews confirmed that the instructions, item content, and response scales were understood by the patients.

**Conclusions:**

Rigorous qualitative research resulted in the development of a PRO measure for MDD with supported content validity. The MDD PRO can assist in understanding and assessing MDD symptoms from patients' perspectives as well as evaluating treatment benefit of new targeted therapies.

## Background

Major Depressive Disorder (MDD) is a highly prevalent and disabling psychiatric illness affecting an estimated 13 to 14 million Americans each year [1-4]. Patients with MDD suffer from persistent depressed mood that adversely affects all aspects of their life - personal, social, and economic. It is associated with a 23-fold increase in the risk of social disability, after controlling for physical diseases [5]. The impact of this disorder also imposes tremendous social and economic burden on society [6]. MDD is the fourth leading cause of disability in the world [7] and is considered to be the mental illness with the largest disease burden on the general population [8].

Patients with MDD show long-term limitations in functioning and well-being that are similar to or worse than those of patients with chronic medical illnesses [9]. Data from the National Institutes of Mental Health (NIMH) Epidemiological Catchment Area Program found that subjects with MDD reported greater financial strain, limitations in physical or occupational functioning, and poorer health status than subjects without MDD [10]. Epidemiologic studies have found that community workers with depression were at least five times more likely to miss work than workers with no symptoms of depression [11,12]. Depressed primary care patients miss two to four more days of work per month due to disability than patients without depression [12].

The diagnosis of MDD, based on the Diagnostic and Statistical Manual of Mental Disorders, Fourth Edition Text Revision (DSM-IV TR), requires: 1) the presence of a single or recurrent major depressive episode (MDE); 2) that MDEs are not better accounted for by schizoaffective disorder and are not superimposed on schizophrenia; and 3) there is no history of manic, mixed, or hypomanic episodes [13]. Thus, the diagnostic action occurs by assessing symptoms that characterize an MDE; these symptoms of depression include depressed mood (such as feelings of sadness or emptiness), reduced interest in activities that were previously enjoyed, weight loss or change in appetite, sleep disturbances, psychomotor agitation or retardation, loss of energy, feelings of guilt or worthlessness, difficulty concentrating, and suicidal thoughts or ideations. Furthermore, a single MDE requires that a patient has experienced at least five of the aforementioned symptoms, one of which must include depressed mood or loss of interest or pleasure, for at least two weeks, and that the symptoms are not a result of the physiological effects of a substance, medical condition, or bereavement. Diagnosis for *recurrent *MDD requires the presence of two or more MDEs in which the episodes are separated by an interval of at least two consecutive months [13].

Severe depression is further categorized as "severe without psychotic features," where patients display most of the diagnostic symptoms accompanied by a marked disability. Patients displaying signs of hallucinations or delusions are categorized as "severe with psychotic features" [13]. It is estimated that about one third of all outpatients diagnosed with depression are severely depressed [14]. The personal, social, and economic impact of MDD worsens as severity of depression increases. Severe depression is associated with increased risk of disability, decreased work productivity, and higher utilization of health care services [8,15,16].

Several clinician rating scales are presently used in assessment of symptoms and management of patients with depression. The Hamilton Depression Rating Scale (HAM-D) and Montgomery-Åsberg Depression Rating Scale (MADRS) are widely used clinician-rated scales. The Inventory of Depressive Symptomatology (IDS); its shortened version, the Quick Inventory of Depressive Symptomology (QIDS) [17]; and the Beck Depression Inventory (BDI-II) are among commonly used patient-reported measures for depression symptoms [18-21]. Many of these instruments were developed without patient input and were derived directly from clinician rating scales, only changing the perspective from clinician to patient. The Food and Drug Administration (FDA), which approves drugs and their labels, has suggested that patient-reported outcomes (PROs) should be developed with input from patients [22]. As defined by the FDA, a PRO is any data reported directly by a patient without interpretation of the patient's response by a clinician or anyone else within the context of the condition or its therapy [22,23]. As health status measures, PROs have been used to assess treatment benefit and monitor change in health status as well as in public health research [24]. Development of a PRO requires rigorous and transparent qualitative research methods, and equally rigorous quantitative methods are required to test its reliability, validity, and responsiveness [22]. In addition, some of the currently used scales, such as the BDI-II, have other limitations with respect to their use to establish treatment benefit; for example, the BDI-II was originally developed as a screener and as such may not be able to detect change; it has a two-week recall period, which may affect its reliability in patient populations with different levels of severity; and the response options are not mutually exclusive, potentially causing problems in scoring.

The profound impact that MDD has on patients' lives underscores the need for additional investigations into symptoms of MDD. The perspective of patients is crucial in identifying what symptoms are relevant to their daily lives and how they are affected by MDD at different levels of severity. The objective of this cross-sectional study was to develop a patient-reported questionnaire that captures the experience of patients with MDD. This PRO was designed to explore the symptoms of MDD; any changes in those symptoms, such as improvement or deterioration; and associated functional status. This article reports the results of this study and introduces a newly developed PRO to monitor and evaluate treatments of MDD.

## Methods

### Research procedure

**Figure 1:**
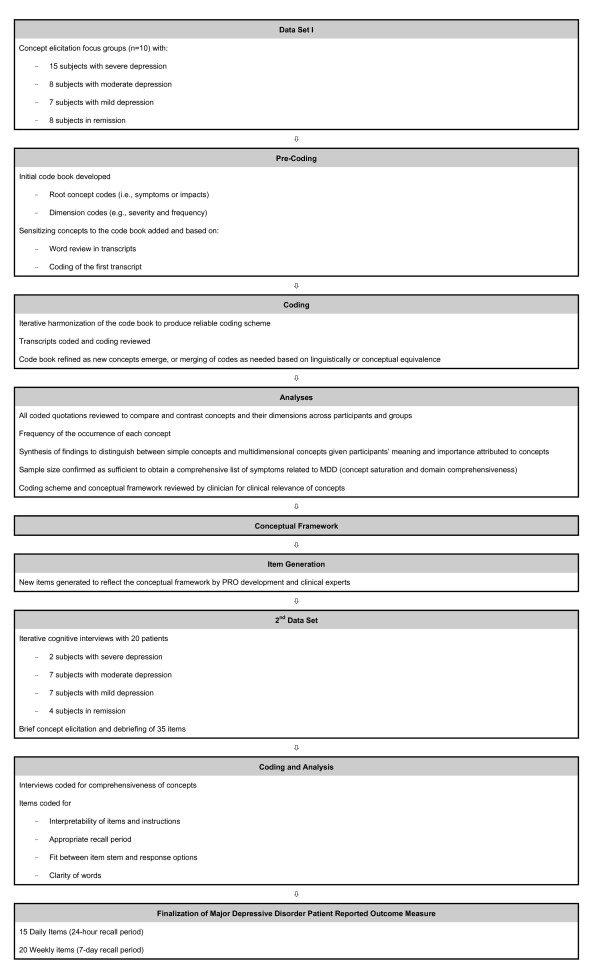
**Qualitative Research Overview**.

Focus groups were conducted based on the principles of grounded theory, which seeks to produce spontaneously elicited, rich descriptions of the symptoms and impacts of MDD. With this approach, the concepts emerge from patients' input, allowing the voice of the patient to be heard rather than applying an *a priori *theoretical model or constructs to interpret the data [25-29]. A focus group methodology was chosen because breadth, rather than depth, of the concepts was desired [24]. An open-ended, semi-structured interview guide was used to generate discussion amongst the focus group members. A typical open-ended question for the focus group guide was, "Please tell me what living with depression means to you." Each focus group, conducted in US English and lasting from 90 to 120 minutes, was homogeneous with regard to severity levels. Each group was facilitated by a moderator and a co-moderator; facilitators included a clinical psychologist who acted as moderator or co-moderator in all focus groups, a medical sociologist with more than twenty years of experience in qualitative research, and trained senior researchers. Mock focus groups between team members were conducted before the actual focus groups, in order to review the focus group guide and to ensure good interviewing practice. From the concepts elicited, a PRO for MDD was developed; PRO items were developed for concepts or themes of depression symptoms that emerged from the qualitative data obtained from the focus groups. The wording for each item was crafted to closely match the original patient-reported data, in order to retain the language patients used to describe their experiences. An 11-point numeric rating scale from "not at all" to "extremely" or "none of the time" to "all of the time" was added to measure each item. This specific response scale was selected due to the ability to detect variability in change over time (responsiveness) using a wider distribution. While not specifically interval in nature, all 11 point NRS allows for distribution-based responsiveness estimations [30]. Figure 1 presents a diagram of the development of the MDD PRO.

### Content validity testing

The new PRO was tested for content validity in cognitive interviews (CIs) that addressed each of the items [31]. CIs were face-to-face, as is standard for this type of interview, where the desired outcome is depth to confirm content validity (as intended by the developer), comprehensibility, relevance, readability, and that the recall period and the fit between item stems and responses are appropriate [22]. Twenty individual in-depth CIs were conducted using open-ended and probing questions. These interviews included a brief, open-ended concept elicitation component to determine the comprehensiveness of concepts important to patients with MDD. All interviewers were trained using Mapi Values' intensive interviewer training regimen. Additional training specific to this study included conducting a mock interview with feedback from the project team. The PRO was cognitively debriefed to determine the understandability, relevance, and interpretability of the items and their response options and any modifications following the results of each iterative set of interviews. A typical question and its probe on the CI interview guide were worded as follows:

Question 1: In the past 24 hours, how irritable have you felt?

1. What does the question mean to you? [Note: If patient repeats the question verbatim, ask the patient to describe an experience.]

a. What does "irritable" mean to you?

To query about response options, the following question was asked during the CIs:

1. What made you choose [Response selected by patient]?

In addition, these interviews assessed the appropriateness of the recall period and the understanding of instructions. First, 10 patient interviews were conducted, after which modifications were made to the PRO. Then this modified version was tested with a second set of five patients and revised once again. Finally this revised version was tested with five additional patients.

All focus group and CIs were transcribed verbatim.

### Participants/patients

Subjects were men and women between the ages of 18 and 65, diagnosed with MDD as defined by the DSM-IV TR criteria, with the aim of capturing patients' experience with episodes of MDD. The study population for both the focus group and CIs was chosen to reflect severity levels to capture any cross-sectional differences between symptoms and function within those levels. The inclusion criteria included: outpatient between the ages of 18 years and 65 years old; a current clinical diagnosis of MDD according to DSM-IV criteria, confirmed by the treating clinician; if diagnosed with comorbid Generalized Anxiety Disorder (GAD), MDD as the primary diagnosis; and fluent US English speaker and willing and able to read, comprehend, and sign an informed consent form. Exclusion criteria included: a lifetime history of DSM-IV Axis I disorder(s) other than MDD, with the exceptions of GAD, comorbid panic disorder, and simple phobias; a DSM-IV Axis II disorder that has a major impact on the patient's current psychiatric status; a DSM-IV Axis II borderline or antisocial personality disorder; a lifetime history of schizophrenia, bipolar, psychosis, or psychotic depression; considered a treatment-refractory patient (defined as having failed adequate courses of treatment with four or more antidepressants or electroconvulsive therapy); a lifetime use of depot antipsychotics; a history of substance or alcohol abuse in the past six months or dependence within the past year (except for caffeine or nicotine dependence), as defined by DSM-IV criteria; currently pregnant or lactating; and cognitive impairment that would interfere with participation in a 90-minute focus group or interview.

"Mini" focus groups were recruited to ensure the size of the focus group would allow all participants to participate and that the groups would not be so large as to be overwhelming to a vulnerable population. Non-probabilistic purposive sampling was used to recruit four focus groups (three to four patients per group) with severe MDD (n = 15), two groups (four patients per group) with moderate depression (n = 8), two groups (three to four patients per group) with mild MDD, and two groups (four patients per group) in remission (n = 8). As there is currently no standardized definition of the varying MDD severity levels, inclusion of subjects across MDD severity level relied on clinician's rating of subjects' current severity level through completion of an overall severity of depression assessment. Clinicians were instructed to classify subjects as having severe, moderate, or mild MDD or as being in remission (partial or full), using the DSM-IV criteria. This information was attached to the Case Report Form completed by the clinician. The treating clinician (a psychiatrist or primary care physician) made the assessment of the severity of MDD.

### Recruitment and institutional review board approval

Subjects were recruited by a commercial agency, Global Market Research Group, through a database of clinicians (including primary care physicians and psychiatrists), who were required to confirm the diagnosis of MDD. Each clinician attributed the subject to one of the five severity groups (i.e., severe, moderate, and mild depression and in partial or full remission) based on a global assessment of condition severity. The Copernicus Group Independent Review Board approved the study and study documents. Each patient had to meet the inclusion and exclusion criteria that were included in the Case Report Form, and each patient was diagnosed by his/her clinician, who signed the Case Report Form. The same procedures were used to recruit and confirm diagnoses for the CIs. Each patient signed an informed consent form and Health Insurance Portability and Accountability Act of 1996 (HIPAA) forms and received a stipend of US$125 for their participation. Subjects were assured of confidentiality through deidentification of the data.

### Setting

Focus groups were conducted at commercial non-clinical and clinical facilities in Philadelphia, New York, Boston, Atlanta, and Chicago. CIs were held at commercial non-clinical facilities in California, Minnesota, and Louisiana. Procedures and interviews guides were consistent across all sites and severity groups.

### Coding and analysis

ATLAS.ti Version 6.0, a computerized qualitative data analysis package, was used to code and analyze the data [28]. A coding scheme including key symptoms and impacts and dimension descriptors (e.g., frequency, severity, duration, pattern, triggers) was iteratively developed by the research team and was modified as analysis progressed to reflect emerging concepts or to merge conceptually equivalent codes [32,33]. Initially, one transcript was coded by all the same project team members who had also conducted the interviews to develop the coding scheme. Harmonization of the coding scheme was accomplished through research team discussion when disputes arose, and this process continued until all transcripts had been coded. Each transcript was independently coded by one of four coders; coding was reviewed by senior members of the team to ensure consistency and reliability of the coding process.

**Figure 2:**
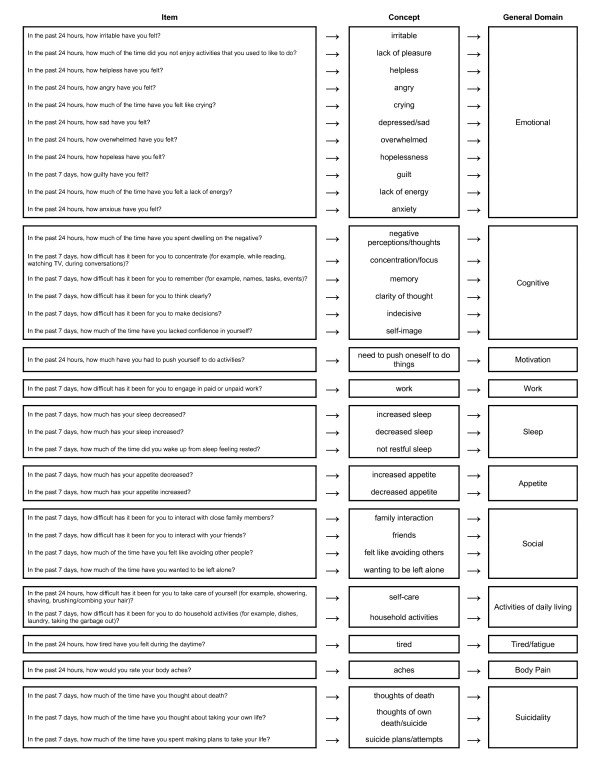
**Conceptual Framework of Major Depressive Disorder Patient-Reported Outcome Measure -Daily and Weekly**.

At the end of the coding process, patterns in the data were determined. Interpretation of the data was performed using a constant comparison method. This process allowed patients' quotes to be compared and contrasted among and between focus groups to identify recurrent concepts relevant to the symptoms and effect of those symptoms on the function of patients with MDD. Concepts that pertained to the same phenomenon were grouped into general domains. See Figure 2 for a conceptual framework showing how items, concepts, and domains are related [22]. To ensure the clinical relevance of concepts in the conceptual framework, the conceptual framework and its development process were reviewed by a clinician (JE). To ensure the adequacy of the sample size and that sufficient data were collected to document elaboration of the concepts and dimensions that constitute the conceptual framework, concept saturation was assessed by documenting concept emergence across sets of successive interviews [26,27]. Concept saturation is achieved when further interviews would be unlikely to elicit new, important, and clinically relevant concepts.

Concepts important to subjects suffering from MDD were identified and compared and contrasted according to MDD severity levels. Concepts that emerged from the focus group discussions highlighted the complex symptomatology of MDD and its associated impact on subjects' health-related quality of life. Due to the nature of mental health disorders, the classification of a spontaneously reported concept as a symptom or impact of MDD was challenging and was achieved only through counsel from practicing clinicians. For example, impaired concentration was initially coded as a symptom of MDD but then reclassified as an impact of MDD, as impaired concentration appeared to be a direct and measurable activity impacted by the symptoms. To distinguish between symptoms and impacts, the research team followed the following definitions: changes in subjective feelings, ideation, impulses, or desires (e.g., "I want to be alone") were identified as symptoms; and changes in psychosocial behavior (e.g., social relationships, work function, functioning in daily routine, etc.) were identified as activities impacted by the symptoms.

## Results

### Study population

Thirty-eight patients were recruited for the concept elicitation study. Forty subjects were scheduled; however, two subjects did not show. One subject left mid-focus group session due to discomfort in the group setting (he was the only male in the group of four); his data, up to the point of his departure, was deemed useful to include in the analysis (the subject also completed the demographic and health information form via mail). Twenty-six women (68%) and 12 men (32%) participated in the concept elicitation interviews. Seventy-six percent of the subjects were Caucasian, and all patients had a high school education or its equivalent. Fifteen subjects were diagnosed with severe, eight with moderate, and seven with mild depression; eight subjects were in remission.

Twenty patients participated in the CIs; their ages ranged from 26 to 74, with a mean age of 44 (standard deviation [SD] = 13.5). Most patients were female (n = 17, 85%) and Caucasian (n = 13, 65%). Eight patients (40%) were working full- or part-time. Two patients were diagnosed with severe, seven with moderate, and seven with mild depression; four patients were in remission. See Table 1 for a more detailed description of the demographic details for the focus groups and CIs and Table 2 for detailed information about their health status.

**Table 1:** Summary of Subjects' Socio-demographic Information

Criteria	Focus Group Population	Focus Group Severity				Content Validation Population	Content Validation Severity			
				
		Severe	Moderate	Mild	In Remission		Severe	Moderate	Mild	In Remission
Sample size	38	15	8	7	8	20	2	7	7	4
Gender (Female)	26 (68.4%)	10	6	4	6	17 (85.0%)	1	6	7	3

Age Mean ± SD (in years) [Range]	47.7 [25-66]	50 [25-66]	44 [34-54]	49 [34-63]	44 [29-56]	43.7 [26-74])	40.5 [35-46])	43.1 [26-52])	44.7 [27-74])	44.3 [31-60])

Ethnicity										

Black/African American	6 (15.8%)	3	0	1	2	2 (10.0%)	1	0	1	0
Hispanic/Latino (of any race)	0 (0%)	0	0	0	0	3 (15.0%)	0	0	2	1
White/Caucasian	29 (76.3%)	11	6	6	6	13 (65.0%)	1	6	4	2
Multiracial	0 (0%)	0	0	0	0	1 (5.0%)	0	1	0	0
Other	0 (0%)	0	0	0	0	1 (5.0%)	0	0	0	1
Missing data	3 (7.9%)	1	2	0	0	0 (0%)	0	0	0	0

Location										

Georgia	8 (21.1%)	4	4	0	0	0 (0%)	0	0	0	0
Massachusetts	8^i ^(21.1%)	0	4	0	4	0 (0%)	0	0	0	0
Illinois	6 (15.8%)	3	0	3	0	5 (25.0%)	1	1	3	0
New York	8 (21.1%)	4	0	4	0	0 (0%)	0	0	0	0
Philadelphia	8 (21.1%)	4	0	0	4	0 (0%)	0	0	0	0
Minnesota	0 (0%)	0	0	0	0	6 (30.0%)	0	4	0	2
Louisiana	0 (0%)	0	0	0	0	5 (25.0%)	0	1	2	2
California	0 (0%)	0	0	0	0	4 (20.0%)	1	1	2	0

Born in U.S.										

Yes	35 (92.1%)	14	6	7	8	19 (95.0%)	2	7	7	3
No	0 (0%)	0	0	0	0	1 (5.0%)	0	0	0	1
Missing data	3 (7.9%)	1	2	0	0	0 (0%)	0	0	0	0

Level of education										

Less than high school	0 (0%)	0	0	0	0	0 (0%)	0	0	0	0
Some high school	0 (0%)	0	0	0	0	0 (0.0%)	1	1	0	1
High school diploma or GED	7 (18.4%)	3	1	1	2	3 (15.0%)	0	1	4	0
Some college	9 (23.7%)	5	2	1	1	5 (25.0%)	0	0	0	0
Vocational school or certificate program^i^	2 (5.3%)	0	1	1	0	0 (0.0%)	1	4	2	1
College or university degree (2- or 4- year)	9 (23.7%)	2	1	3	3	8 (40.0%)	0	0	0	2
Graduate degree	7 (18.4%)	3	1	1	2	2 (10.0%)	0	1	1	0
Missing data	4 (10.5%)	2	2	0	0	2 (10.0%)	0	0	0	0

Work status										

Full-time or part-time	16 (42.1%)	3	5	5	3	8 (40.0%)	1	2	2	3
Full-time or part-time homemaker	0 (0%)	0	0	0	0	4 (20.0%)	0	2	1	1
Unemployed	10 (26.3%)	6	0	1	3	3 (15.0%)	0	1	2	0
Student	0 (0%)	0	0	0	0	1 (5.0%)	0	1	0	0
Retired	1 (2.6%)	1	0	0	0	1 (5.0%)	0	0	1	0
Other (disabled)	3 (7.9%)	1	1	1	0	2 (10.0%)	1	1	0	0
Other (social security)	2 (5.3%)	0	0	0	2	0 (0%)	0	0	0	0
Other (unspecified)	2 (5.3%)	2	0	0	0	0 (0%)	0	0	0	0
Missing data	4 (10.5%)	2	2	0	0	2 (10.0%)	0	1	1	0

Relationship status										

Single	15 (39.5%)	8	1	3	3	3 (15.0%)	1	0	2	0
Significant other	3 (7.9%)	0	2	0	1	3 (15.0%)	0	0	2	1
Married	9 (23.7%)	3	1	3	2	11 (55.0%)	1	6	2	2
Separated	1 (2.6%)	0	0	0	1	0 (0.0%)	0	0	0	0
Divorced	6 (15.8%)	2	2	1	1	2 (10.0%)	0	0	1	1
Widowed	0 (0%)	0	0	0	0	0 (0.0%)	0	0	0	0
Other	0 (0%)	0	0	0	0	0 (0.0%)	0	0	0	0
Missing data	4 (10.5%)	2	2	0	0	2 (10.0%)	0	1	1	0

Living situation										

Alone	16 (42.1%)	8	1	3	4	1 (5.0%)	0	0	0	1
With family (immediate or extended)	9 (23.7%)	3	2	2	2	12 (60.0%)	1	4	5	2
With a friend(s) or roommate(s)	3 (7.9%)	1	2	0	0	1 (5.0%)	1	0	0	0
With significant other	6 (15.8%)	1	1	2	2	5 (25.0%)	0	2	1	2
At a nursing home	0 (0%)	0	0	0	0	0 (0.0%)	0	0	0	0
Assisted living	0 (0%)	0	0	0	0	0 (0.0%)	0	0	0	0
Other	0 (0%)	0	0	0	0	0 (0.0%)	0	0	0	0
Missing data	4 (10.5%)	2	2	0	0	2 (10.0%)	0	1	1	0

^i^: One patient dropped out of study during focus group

Note: Percentages (%) are rounded; each criterion (where appropriate) ≈ 100%.

**Table 2:** Summary of Subjects' Health Information

Criteria	Focus Group Population	Focus Group Severity				Content Validation Population	Content Validation Severity			
				
		Severe	Moderate	Mild	In Remission		Severe	Moderate	Mild	In Remission
Sample size	38	15	8	7	8	20	2	7	7	4
Health in general										

Excellent	1 (2.6%)	0	0	0	1	4 (20.0%)	1	0	2	1
Very good	8 (21.1%)	1	3	2	2	5 (25.0%)	0	3	2	0
Good	12 (31.6%)	6	0	2	3	7 (35.0%)	0	3	2	2
Fair	11 (28.9%)	6	3	1	1	3 (15.0%)	1	0	1	1
Poor	3 (7.9%)	1	0	1	1	1 (5.0%)	0	1	0	0
Missing data	3 (7.9%)	1	2	0	0	0 (0%)	0	0	0	0

Comorbid conditions										

Anxiety disorder	23 (60.5%)	12	3	4	4	9 (45.0%)	0	4	4	1
Arthritis	7 (18.4%)	4	0	2	1	2 (10.0%)	0	1	0	1
Cancer	1 (2.6%)	0	0	1	0	1 (5.0%)	0	1	0	0
Chronic headaches	4 (10.5%)	3	1	0	0	4 (20.0%)	0	1	2	1
Chronic low back pain	9 (23.7%)	5	2	0	2	6 (30.0%)	1	2	3	0
Colitis	0 (0%)	0	0	0	0	0 (0.0%)	0	0	0	0
Diabetes	3 (7.9%)	1	1	0	1	2 (10.0%)	0	1	0	1
Fibromyalgia	1 (2.6%)	1	0	0	0	1 (5.0%)	0	0	1	0
Heart or circulatory condition	1 (2.6%)	0	0	1	0	3 (15.0%)	0	1	1	1
Interstitial cystitis	0 (0%)	0	0	0	0	0 (0.0%)	0	0	0	0
Panic disorder	7 (18.4%)	4	1	1	1	3 (15.0%)	0	0	2	1
Thyroid disease	1 (2.6%)	1	0	0	0	2 (10.0%)	0	2	0	0
None	6 (15.8%)	2	3	0	1	4 (20.0%)	1	0	1	2
Other	9 (23.7%)	4	0	3	2	2 (10.0%)	0	0	1	1
Missing data	0 (0%)	0	0	0	0	1 (5.0%)	0	0	1	0

Type of care^ii^										

Care from a psychiatrist	31 (81.6%)	14	4	5	8	4 (20.0%)	1	2	0	1
Care from a psychologist	14 (36.8%)	8	4	2	5	4 (20.0%)	1	0	2	1
Care from a primary physician	12 (31.6%)	4	2	3	3	18 (90.0%)	1	7	6	4
Mental health clinic	5 (13.2%)	3	2	0	1	1 (5.0%)	0	0	0	1
Support group	7 (18.4%)	4	2	1	0	0 (0%)	0	0	0	0
Other (Therapist)	2 (5.3%)	0	0	0	2	0 (0%)	0	0	0	0
Other (NP)	1 (2.6%)	0	1	0	0	0 (0%)	0	0	0	0
Other (Hospital)	0 (0%)	0	0	0	0	1 (5.0%)	0	0	1	1
Other (Medication)	0 (0%)	0	0	0	0	1 (5.0%)	0	0	1	1
Missing data	3 (7.9%)	1	2	0	0	0 (0%)	0	0	0	0

Medication^ii^										

SSRI	17 (44.7%)	7	0	6	4	12 (60.0%)	1	3	6	2
NDRI	4 (10.5%)	3	1	0	0	3 (15.0%)	1	2	0	0
SNRI	12 (31.6%)	6	2	1	3	2 (10.0%)	0	0	1	1
Tricyclic	2 (5.3%)	1	0	0	1	0 (0.0%)	0	0	0	0
Tetracyclic	2 (5.3%)	1	0	1	0	0 (0.0%)	0	0	0	0
Atypical antipsychotic	5 (13.2%)	2	1	0	2	0 (0.0%)	0	0	0	0
Other (various)	10 (26.3%)	1	2	2	5	4 (20.0%)	0	2	1	1
Missing data	5 (13.2%)	2	3	0	0	1 (5.0%)	0	0	0	1

Years experiencing symptoms										

< 1 year	1 (2.6%)	0	1	0	0	1 (5.0%)	0	0	0	0
1-5 years	6 (15.8%)	3	0	1	2	13 (65.0%)	0	0	0	0
5-10 years	4 (10.5%)	1	0	2	1	2 (10.0%)	0	0	0	0
10-15 years	6 (15.8%)	2	2	1	1	3 (15.0%)	0	0	0	0
15-20 years	3 (7.9%)	2	0	1	0	0 (0.0%)	0	0	0	0
> 20 years*	13 (34.2%)	5	2	2	4	0 (0.0%)	0	0	0	0
Missing data	5 (13.2%)	2	3	0	0	1 (5.0%)	0	0	0	0

^i^: One subject dropped out of study during focus group

^ii^: Subjects were instructed to check all that apply; total will not equal 100%

*: Subjects who described how long they experienced their symptom with words such as: "forever," "most of my life," "many years," "very long time," etc. are included in this category

Note: Percentages (%) are rounded; each criterion (where appropriate) ≈ 100%x'

### Concepts elicited and development of MDD PRO

Sixty seven (67) distinct concepts were elicited, analyzed, and merged based on conceptual equivalence (e.g., anxiety and panic) and clinical validity (e.g., obsessive and negative thoughts). The final list included 35 concepts that were grouped into 11 general domains. These included: i) emotional (11 concepts such as angry and helpless), ii) cognitive (six concepts including memory and clarity of thought), iii) motivation, iv) work, v) sleep (three concepts), vi) appetite (two concepts), vii) social (four concepts), viii) activities of daily living (two concepts), ix) tired/fatigue, x) body pain, and xi) suicidality (three concepts). All concepts but three (i.e., engage in intimacy, ability to articulate, and clear-headed) were saturated. Items were generated to reflect each of the 35 concepts. These concepts resulted in 15 daily and 20 weekly items, (i.e., recall periods of the "past 24 hours" and "past seven days," respectively). Thirty-two items used an 11-point severity or frequency numerical rating scale response format. One item asked for the number of hours of sleep in the past 24 hours, and one item asked, "How would you rate the amount of sleep you had in the past twenty-four hours?" with the responses, "less than I would have liked," "about the right amount," and "more than I would have liked." One item asked "How would you rate the amount you have eaten in the past twenty-four hours?" with responses, "less than I would have liked," "about the right amount," or "more than I would have liked."

Four of the most frequent concepts reported were "avoidant" (n = 34), "self-image/confidence" (n = 30), "thoughts of death" (n = 25) and "suicidal ideation" (n = 25). Subjects across all severity groups avoided others when depressed. Subjects used the words, "isolate," "tune out," "not a people person," "hide from the world," "shut people out," "shut down," "alienation," "world gets smaller," "avoidance," "hibernated state," "solitaire [*sic*]," and "withdrawn." Subjects described the need to avoid others in the following ways:

"I shut everybody out. I pick up my book, my book goes in front of my face, and I don't want to hear anybody, don't talk to me... Leave me alone and let me live in my own little world for now. And that's what I do when I shut down" (severe).

I actually isolate myself. I isolate myself. I become very withdrawn. I don't leave. I don't interact. I don't want to go out. I don't want to deal with anyone (mild).

When discussing their avoidance, subjects described themselves as avoiding people or situations, hiding, sleeping, or staying in bed, avoiding self-care, avoiding emotions, feeling safe at home, and losing communication with people, saying, for example:

It's a very dark place... You do fall out of communication with people and you just kind of sit in bed and lack the willpower to really get out there and do what you need to do (moderate).

When describing a worst day with depression, subjects indicated their need to be left alone, to avoid any interactions or situations prompting interaction ("picking up the phone,"). One patient, for example, reported that when depressed:

"I don't feel like getting out of bed and facing the world. I don't feel like eating, I feel like burying my head in the sand like an ostrich. And I just don't feel like - I'm just nervous around people. I don't want to be around them" (remission).

Subjects were candid about suicide. Nine subjects in three severe groups, both moderate groups, and one each of the mild and remission groups discussed their experiences with suicide attempts, describing the various methods attempted (e.g., wrist cutting, swallowing pills, hanging self). At the time of their suicide attempt, patients saw death as their "only option," saying, for example, "At those times it just... seem like there was no way out" (mild) and "I finally decided that I just wanted to give up, and I tried to take my life (severe).

MDD is described by subjects as a constellation of symptoms, including depressed mood (e.g., feeling sad), and impacts. The complex, cluster-like presentation of MDD became evident through subjects' descriptions of symptoms as impacts and vice versa (e.g., decreased concentration as a symptom and/or impact of MDD). Following the analysis of the transcripts from the focus groups, the comprehensive list of symptoms and impacts spontaneously mentioned by subjects with MDD was reviewed and organized into a conceptual framework (available upon request). A draft patient-completed instrument was developed to assess the symptoms and functional impairments associated with MDD. While there are currently several clinician-completed tools for diagnosis and management of MDD, few, if any, capture the patients' perspective.

### Cognitive interviews of the MDD PRO

The first round of CIs of the 35-item questionnaire found the items understandable, relevant, and readable on the whole across severity groups. Several patients commented on the difficulty of using a 24-hour recall period for concepts such as avoiding other people and those which involved activities that may not occur every day. Some items were changed from daily to weekly items due to patient input that the symptom required a longer recall period to capture variation and frequency. This resulted in development of a daily and weekly PRO questionnaire. Because both a daily and a weekly recall period were tested, patients were specifically asked about the appropriateness of the recall period for each concept presented. The choice of placing an item into the daily or weekly questionnaire was a direct result of the patients' preference (relevance to day-to-day life) and ability to recall changes which may occur with each concept over 24 hours or seven days.

The results of the second round of interviews were similar to the first in terms of understandability, relevance, and readability, with added feedback regarding the questions related to sleep and appetite changes. The results of the third round of interviews were similar to previous rounds, with few changes suggested except for changing some daily items to weekly recall. Patients did suggest, for example, that the item regarding losing interest in just about everything be changed to be more specific (i.e., losing interest in just about all activities) and that the recall period be changed from daily to weekly for this item. For example, one patient stated, "Well, I guess lost interest in just about everything - I'm just trying to think about what everything would be. I guess I just was a little confused at that." Another patient indicated this question was difficult to answer because "... I think it takes more than 24 hours to just lose interest in everything." The 11-point NRS, instructions, and recall period were confirmed in the last set of CIs.

Patients experienced and recognized the emotional concepts (irritability, lack of pleasure, helplessness, anger, crying, sadness, feeling overwhelmed, hopelessness, guilt, lethargy, and anxiety), and felt they were well represented in the questionnaires. Specific probing indicated that although patients defined "helpless" and "hopeless" differently, their initial presentation as adjacent items led to some confusion; moving the items further apart eliminated the confusion between the two words. The concept of "lack of pleasure" was tried in a number of item formats, with one item "how much of the time have you felt that you did not enjoy activities that you used to like to do?" being chosen as the most preferred wording. Other suggestions for wording were incorporated in order to use patient words as much as possible.

The cognitive concepts (negative perceptions/thoughts, problems with concentration/focus, memory problems, clarity of thought, indecisiveness, and lack of confidence) were also commonly experienced by patients and accepted as important to the questionnaires. Specific wording from patients helped to clarify the item on negative thoughts, with "dwell on the negative" being well accepted and understood to represent the concept. Suggestions for parenthetical examples for the concentration and memory items were made by patients, and the changes were confirmed by later CIs and led to patients interpreting the items as intended.

The concepts of changes in eating and sleeping proved more difficult to approach in the versions of the questionnaire tested, since different patients can have different presentations, with either increased or decreased sleeping or eating.

In summary, all daily items were changed after Round 1 to include the phrase, "In the past twenty-four hours," at the beginning of the question rather than the end to focus attention on the recall period; likewise all weekly items were also revised to include the phrase "In the past 7 days" at the beginning of the question. Instructions were changed after Rounds 1 and 2 to indicate the correct number of items in the questionnaire as items were deleted. Six questions were moved from the daily to the weekly recall period as patients' reports indicated this recall was more appropriate. Other changes were relatively minor, such as rephrasing sentence wording to more accurately reflect patients' experience or patients' words. For example, "In the past 7 days, how difficult has it been for you to interact with close relatives?" was changed to, "In the past 7 days, how difficult has it been for you to interact with close family members?" and "energetic" was changed to "lack of energy." In addition, the order of some items was changed, such as moving the items "helpless" and "hopeless" further apart in the questionnaire after patient input suggested their proximity may be lead to confusion between the terms. Finally, separate questions on "increases" and "decreases" in appetite and sleep were created instead of using "changes" in appetite or sleep.

## Discussion

This MDD questionnaire is the first to date to be developed through patient input in accordance with the FDA's *Guidance for Industry: Patient-Reported Outcome Measures *[22]. The research reported here produced data of high quality, given the rigorous data collection, management, and analytic procedures, and is of importance to the field of psychiatry because it allows a window to witness the experiences of patients suffering from a very debilitating disorder from the patient's voice and perspective. The content validity of the MDD PRO was supported through analysis of both the focus group and CI data; further support of its content validity, however, is required by means of psychometric validation.

The design of this study, to start with focus groups where the intent is to elicit breadth of concepts and to conclude with individual in-depth CIs, allowed the content validity of the PRO MDD to be supported. This study showed that patients with MDD are able to participate in studies of this type, supporting the feasibility of their responding to a questionnaire that can monitor changes in their condition and that can be used to evaluate treatments. Only one person had to leave the focus group due to the stress of this experience, and all participants but two showed up for their group. A further strength of this study is that the experience of MDD was captured across severity levels, including mild, moderate, severe, and in remission (partial and full), in order to produce a measure that could capture worsening, improvement, or the lack of change. Because few PRO measures for depression have been developed with documented patient input, a strong suit of this instrument is that the exact words patients use to describe the symptoms of MDD and their duration, frequency, and severity, were elicited, as were the appropriate recall periods for these symptoms. Items derived from these concepts were tested during cognitive interviews. As mentioned in the introduction, the importance of the patient's voice in the development of questionnaires to measure treatment benefit increasingly has been emphasized and made public.

While this study has many strengths, one limitation of this study is that the same group of patients was not followed over time through episodes of major depression. Although this cross-sectional approach of looking at severity levels was efficient and in line with cost constraints, future research can use this instrument to determine if it is responsive to changes in mental health status over time. Future work should also address its construct validity and its test-retest, and internal consistency reliability. A further limitation is that the sample is relatively highly educated; no patients in the CE or CI interviews had less than a high school education. There are no Hispanic or Latino patients in the focus groups and no Native Americans; the absence of Native Americans, however, may have been a function of the cities from which we recruited, among other factors. It is to be noted, however, that there was 7.9% missing data for ethnicity in the focus group sample. The ethnic distribution of patients is more representative in the content validity testing sample, which may attenuate any potential bias. One solution to this lack of representativeness would be to test the content validity, validity, and reliability of this instrument in diverse cultural groups.

## Conclusion

The MDD PRO was developed with rigorous qualitative research, and this research has supported its content validity. Once it has undergone psychometric testing, it can assist in recognizing MDD in patients of primary care physicians, as well as in evaluating treatment benefit of new targeted therapies. The MDD PRO has 35 items, 15 of which have a 24-hour recall period; the remaining items have a seven-day recall period. Both recall periods were suggested in the concept elicitation interviews and confirmed in the CIs. The MDD PRO is a much-needed addition to measuring patient-reported outcomes in patients suffering from this disabling condition.

## Competing interests

This project was supported by funding from AstraZeneca Pharmaceuticals LP. Kathryn Eilene Lasch, Elisabeth Carine Piault-Luis, Marcy Fitz-Randolph, Steve Hwang, Kasey Jernigan are or were employees of Adelphi Values during the conduct of this study and write-up of this manuscript. Elizabeth Carine Piault-Luis is currently a consultant for Adelphi Values and Kasey Jernigan is in a doctoral program at the University of Massachusetts, Amherst. Mariam Hassan, Julie Locklear, and Sanjeev Pathak are employees of Astra Zeneca. Jean Endicott is an employee of Columbia University and was retained as a clinical expert during the conduct of this study by Adelphi Values.

## Authors' contributions

KL, MH, JE, EP-L, and JL contributed to the design and overall conduct of the study. KL drafted the manuscript. MH, JE, EP-L, SP, and JL reviewed the manuscript draft critically and made important contributions to its revision. SP and JE provided significant contribution in terms of the medical knowledge required for this study throughout. KL, MF-R, SH and KJ contributed to the acquisition, coding, and analysis of data as well as to interpretation of results. All authors contributed to the interpretation of results. All authors contributed to the design of the PRO measure and its revisions. All authors read and approved the final manuscript.

## Pre-publication history

The pre-publication history for this paper can be accessed here:

http://www.biomedcentral.com/1471-244X/12/34/prepub
